# Supplementary material for: Neural representation of newly instructed rule identities during early implementation trials
Source: eLife. 2019 Nov 18;8:e48293. doi: 10.7554/eLife.48293 (PMC6884394; doi:10.7554/eLife.48293)
Supplement: Transparent reporting form [file elife-48293-transrepform.docx]

***eLife’s* transparent reporting form**

We encourage authors to provide detailed information *within their submission* to facilitate the interpretation and replication of experiments. Authors can upload supporting documentation to indicate the use of appropriate reporting guidelines for health-related research (see [EQUATOR Network](http://www.equator-network.org/%20)), life science research (see the [BioSharing Information Resource](https://biosharing.org/)), or the [ARRIVE guidelines](http://www.plosbiology.org/article/info:doi/10.1371/journal.pbio.1000412) for reporting work involving animal research. Where applicable, authors should refer to any relevant reporting standards documents in this form.

If you have any questions, please consult our Journal Policies and/or contact us: [editorial@elifesciences.org](mailto:editorial@elifesciences.org).

**Sample-size estimation**

- You should state whether an appropriate sample size was computed when the study was being designed
- You should state the statistical method of sample size computation and any required assumptions
- If no explicit power analysis was used, you should describe how you decided what sample (replicate) size (number) to use

Please outline where this information can be found within the submission (e.g., sections or figure legends), or explain why this information doesn’t apply to your submission:

Given the excessive multiple comparison problem in functional MRI analyses we primarily adopted a ROI-based analysis approach which was complemented by a secondary voxel-wise whole-brain analysis approach. The primary statistical analyses were based on a small number of anatomically-defined ROIs (left/right VLPFC and DLPFC). In order to minimize multiple ROI testing their number was kept as small as possible focusing on the theoretically most relevant regions. Sample size calculation (using Gpower 3.1.9.4) was based on the requirements for a paired t-test with (1 – type II error probability) = .9, type I error probability =.05 (two-tailed, uncorrected), assuming an effect size within the medium range (dz=.4). This resulted in a sample size of N=68. For secondary voxel-wise whole-brain analyses this sample size allowed us to detect large effects (dz=.75) with (1 – type II error probability) = .85, and type I error probability = 0.00001 (two-tailed, uncorrected; this equals a critical t=4.8 which is in the range of typical t values required for FWE-correction at the whole-brain level).

**Replicates**

- You should report how often each experiment was performed
- You should include a definition of biological versus technical replication
- The data obtained should be provided and sufficient information should be provided to indicate the number of independent biological and/or technical replicates
- If you encountered any outliers, you should describe how these were handled
- Criteria for exclusion/inclusion of data should be clearly stated
- High-throughput sequence data should be uploaded before submission, with a private link for reviewers provided (these are available from both GEO and ArrayExpress)

Please outline where this information can be found within the submission (e.g., sections or figure legends), or explain why this information doesn’t apply to your submission:

- Each experiment was performed once.
- There were no outliers
- Data from 5 (out of 140) subjects were excluded due to early abortion during scanning (page 22)

**Statistical reporting**

- Statistical analysis methods should be described and justified
- Raw data should be presented in figures whenever informative to do so (typically when N per group is less than 10)
- For each experiment, you should identify the statistical tests used, exact values of N, definitions of center, methods of multiple test correction, and dispersion and precision measures (e.g., mean, median, SD, SEM, confidence intervals; and, for the major substantive results, a measure of effect size (e.g., Pearson's r, Cohen's d)
- Report exact p-values wherever possible alongside the summary statistics and 95% confidence intervals. These should be reported for all key questions and not only when the p-value is less than 0.05.

Please outline where this information can be found within the submission (e.g., sections or figure legends), or explain why this information doesn’t apply to your submission:

- Statistical analysis methods are described in the Methods: pages 25-29.
- No raw data included in Figures: N was >10
- Mean, CI, p-values, and effect sizes are reported.

(For large datasets, or papers with a very large number of statistical tests, you may upload a single table file with tests, Ns, etc., with reference to sections in the manuscript.)

**Group allocation**

- Indicate how samples were allocated into experimental groups (in the case of clinical studies, please specify allocation to treatment method); if randomization was used, please also state if restricted randomization was applied
- Indicate if masking was used during group allocation, data collection and/or data analysis

Please outline where this information can be found within the submission (e.g., sections or figure legends), or explain why this information doesn’t apply to your submission:

No group comparison was done

**Additional data files (“source data”)**

- We encourage you to upload relevant additional data files, such as numerical data that are represented as a graph in a figure, or as a summary table
- Where provided, these should be in the most useful format, and they can be uploaded as “Source data” files linked to a main figure or table
- Include model definition files including the full list of parameters used
- Include code used for data analysis (e.g., R, MatLab)
- Avoid stating that data files are “available upon request”

Please indicate the figures or tables for which source data files have been provided:

Preprocessed single subject data and whole-brain maps underlying the main results visualized in Fig. 4, Fig. 5, Fig. 6, Fig. 7, and Fig. 8 are provided here:

https://osf.io/vsbx8/
